# Non-Pharmacological Interventions to Reduce Unhealthy Eating and Risky Drinking in Young Adults Aged 18–25 Years: A Systematic Review and Meta-Analysis

**DOI:** 10.3390/nu10101538

**Published:** 2018-10-18

**Authors:** Stephanie Scott, Fiona Beyer, Kathryn Parkinson, Cassey Muir, Alice Graye, Eileen Kaner, Martine Stead, Christine Power, Niamh Fitzgerald, Jen Bradley, Wendy Wrieden, Ashley Adamson

**Affiliations:** 1 School of Social Sciences, Humanities and Law, Teesside University, Middlesbrough TS1 3BA, UK; 2 Fuse—The Centre for Translational Research in Public Health, a UK Clinical Research Collaboration (UKCRC) Public Health Research Centre of Excellence, Newcastle upon Tyne NE2 4AX, UK; eileen.kaner@ncl.ac.uk (E.K.); wendy.wrieden@ncl.ac.uk (W.W.); ashley.adamson@ncl.ac.uk (A.A.); 3 Institute of Health & Society, Baddiley-Clark Building, Newcastle University, Richardson Road, Newcastle upon Tyne NE2 4AX, UK; fiona.beyer@ncl.ac.uk (F.B.); kathryn.parkinson@ncl.ac.uk (K.P.); cassey.muir@ncl.ac.uk (C.M.); a.graye@newcastle.ac.uk (A.G.); jen.bradley@ncl.ac.uk (J.B.); 4 Human Nutrition Research Centre, Newcastle University, Newcastle upon Tyne NE2 4AX, UK; 5 Institute for Social Marketing (ISM), Faculty of Health Sciences and Sport, University of Stirling, Stirling FK9 4LA, UK; martine.stead@stir.ac.uk (M.S.); niamh.fitzgerald@stir.ac.uk (N.F.); 6 Population, Policy and Practice, UCL Great Ormond Street Institute of Child Health, 30 Guilford Street, London WC1N 1EH, UK; christine.power@ucl.ac.uk

**Keywords:** Intervention, young adult, eating behaviour, alcohol, systematic review

## Abstract

Alcohol use peaks in early adulthood and can contribute both directly and indirectly to unhealthy weight gain. This review aimed to systematically evaluate the effectiveness of preventative targeted interventions focused on reducing unhealthy eating behavior and linked alcohol use in 18–25-year-olds. Twelve electronic databases were searched from inception to June 2018 for trials or experimental studies, of any duration or follow-up. Eight studies (seven with student populations) met the inclusion criteria. Pooled estimates demonstrated inconclusive evidence that receiving an intervention resulted in changes to self-reported fruit and vegetable consumption (mean change/daily servings: 0.33; 95% CI −0.22 to 0.87) and alcohol consumption (mean reduction of 0.6 units/week; CI −1.35 to 0.19). There was also little difference in the number of binge drinking episodes per week between intervention and control groups (−0.01 sessions; CI −0.07 to 0.04). This review identified only a small number of relevant studies. Importantly, included studies did not assess whether (and how) unhealthy eating behaviors and alcohol use link together. Further exploratory work is needed to inform the development of appropriate interventions, with outcome measures that have the capacity to link food and alcohol consumption, in order to establish behavior change in this population group.

## 1. Introduction

Excess body weight and heavy alcohol consumption are two of the greatest contributors to global disease burden [1,2]. There is now an established and well-evidenced relationship between overweight/obesity and alcohol use in adulthood. Body mass index (BMI) and alcohol consumption interact, with a steeply elevated risk of liver disease observed for those with high BMI and alcohol levels [3]. Heavy drinking is also associated with greater waist-hip-ratio in mid-life even when taking other lifetime influences into account [4]. Thus, strategies that aim to jointly reduce alcohol consumption and address levels of overweight or obesity may produce greater health gains, and be a more efficient use of resources, than initiatives directed towards each behavioral pattern alone. Alcohol use remains the leading cause of death and disability adjusted life years in both 15–19-year-olds and 20–24-year-olds globally [5]; whilst young adults consume, on average, less fruit and fewer vegetables than the minimum recommendation [6]. Meanwhile, young adults also tend to prioritize foods that are convenient, taste good, and are low in cost [7], many of which can be defined as energy-dense, highly-processed foods and drinks.

A growing body of epidemiological data has identified that a relationship between alcohol use and unhealthy eating behaviors begins as early as adolescence and young adulthood. Energy intake from alcohol, type of beverage, and drinking pattern (i.e., high volume, high frequency) contribute substantially to dietary energy intake and are associated with excess body weight and weight gain amongst young adults [8,9]. Further, regular heavy episodic drinking in young adulthood is associated with higher risk of transitioning to being overweight or obese [9]. For example, young adults with high levels of alcohol consumption on a single occasion are more likely to be obese than those with a lower intake (up to 25% Recommended Daily Allowance (RDA) Energy) (Albani et al., in press). As a result, some individuals may choose not to eat prior to socializing, so that they can drink alcohol and avoid weight gain; a phenomenon that has been termed ‘Drunkorexia’, and is reported to be particularly prevalent among US college students [10,11,12,13]. Unhealthy weight-control methods linked to problematic alcohol use such as this can emerge as early as mid-adolescence [14], with some individuals conflicted by a wish to stay slim, but also to drink alcohol as part of developing a social identity [10,15,16]. Practices which restrict energy intake prior to alcohol consumption increase the likelihood of intoxication, where blood alcohol levels rise sharply and affect the brain and subsequent behavior, steeply increasing the risk of acute harm from drinking, such as from accidents or becoming a victim of crime.

In certain drinking cultures, many eating rituals are strongly linked to the use of alcohol and vice versa: For instance, salty snacks are sold in public drinking venues, and it is common to drink alcohol with a meal or visit fast food outlets after an evening out at drinking establishments [17]. Unhealthy food choices are more likely during and directly after a drinking occasion [17], due in part to the disinhibiting effect of alcohol, which is a psychoactive substance that can alter usual behavior. Whilst linked, there are key differences when considering eating behaviors and alcohol use. Food is essential for survival whilst alcohol is not. Alcohol contains energy, but it is a nutritionally poor food source and does not stimulate satiety [18]. Nevertheless, for many, both are a source of pleasure and a valued component of social life. However, the links between unhealthy eating behavior and risky alcohol use in the social, emotional and cultural lives of young adults remains under-studied (Scott et al., under review). Instead, research focusing on the reduction of excess body weight or heavy alcohol consumption typically occurs in isolation, or as part of non-specific multiple behavior change interventions. Recent exceptions have been behavioral modification work with middle aged men with obesity who were also alcohol drinkers [19,20], and the BeWEL study which sought to reduce weight by addressing diet, physical activity, and alcohol among middle aged adults at increased risk of colorectal cancer and other obesity related comorbidities [21]. In the latter study, attention paid to alcohol was less than that paid to diet and physical activity.

Whilst a published Cochrane systematic review examines individual, family, and school-led interventions for preventing multiple risk behaviors in individuals aged eight to 25 years [22], risk tends to be focused upon smoking, drinking and/or drug taking. To our knowledge, no systematic review has examined the impact of interventions to reduce unhealthy eating and linked risky drinking among adults or individuals aged 18–25 years. Therefore, it is unknown whether interventions of this nature are especially efficacious or whether there is a proliferation of interventions in this area. Thus, our review aimed to address this evidence gap by systematically evaluating the current evidence-base on the effectiveness of preventative targeted interventions focused on reducing unhealthy eating behavior and linked risky alcohol use in young adults aged 18–25 years.

## 2. Materials and Methods 

This systematic review and meta-analysis protocol was pre-registered (CRD42016040128) [23] in compliance with the ‘Preferred Reporting Items for Systematic Reviews and Meta-Analyses’ (PRISMA) Statement [24,25].

### 2.1. Eligibility Criteria

The following studies were eligible for inclusion: randomised controlled trials (RCTs), including cluster RCTs; non-randomized controlled trials (e.g., studies with multiple clusters/communities where allocation to interventions was not randomized); interrupted time series; quasi-experimental; cohort involving concurrent or historical controls; and controlled before and after studies of non-pharmacological interventions targeted at free-living (not mandated, hospitalized or imprisoned) male and female young adults aged 18–25 years (or where the mean age of participants fell within this range) in any country, and whose linked outcomes were assessed for this group. Primary outcomes of interest were reported changes in: (1) Dietary, nutritional or energy intake, and (2) alcohol consumption. Secondary outcomes were measures of body composition and alcohol-related outcomes which do not focus directly on consumption (i.e., BMI, waist circumference, waist-hip ratio, % body fat, biochemical measures, purchasing behavior, and hospital admissions). We focused on targeted interventions to improve dietary and alcohol consumption behavior at an early stage of risk or harm, when it is likely to be most amenable to change [26]. We defined this to include the identification of relevant individuals, followed by the delivery of behavior modification strategies (at the individual, community, and societal level) based on information, advice and counselling targeting unhealthy eating and linked risky alcohol use [27,28]. Primarily (but not exclusively) of interest were studies where both behaviors were addressed simultaneously using an outcome measure with the capacity to link these behaviors together, such as energy (calorie) intake (multicomponent interventions). Studies where interventions addressed both behaviors, but measured outcomes separately, were also eligible for inclusion in this review. Differences in measurement were considered when interpreting the review findings. Interventions were compared to control (no intervention or waiting list), assessment only and treatment as usual. Eligible interventions could be delivered in-person, by telephone, by internet, or a combination of multiple delivery methods; individually, as part of a group, or a combination in any setting. There were no restrictions in terms of length of intervention or follow up. Studies were excluded if the study population required specialist treatment for alcohol dependence or weight loss and gain (i.e., bariatric surgery) or if current eating behaviors were time-limited and not reflective of usual dietary behaviors (i.e., pregnant or breastfeeding women).

### 2.2. Search Strategy

We searched electronic databases (MEDLINE, Embase, PubMed, PsycINFO, ERIC, ASSIA, Web of Knowledge, Scopus, CINAHL, LILACS, CENTRAL, and ProQuest Dissertations and Theses) without language restriction, from inception to June 2018, using appropriate thesaurus headings and title or abstract keywords. The search strategy for the review is documented in Supplementary Table S1. Thesaurus terms were translated as appropriate across databases. Database searches were supplemented with searches of trial registers (World Health Organization International Clinical Trials Registry; Meta-Register of Controlled Trials), searches of Google Scholar for relevant studies using the names of authors of included papers; and hand searching of the reference lists of included papers and relevant reviews.

### 2.3. Study Selection and Data Extraction

**Figure 1:**
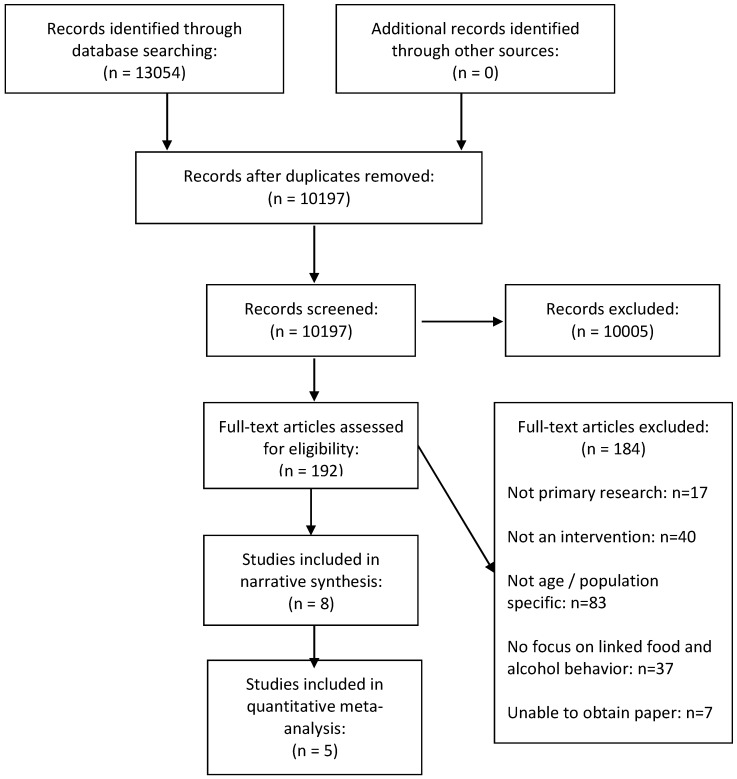
Flow chart showing study selection process.

The title and abstract of all records retrieved were downloaded to Endnote X7 and independently screened by four reviewers (SS, KP, CM, AG), with full text copies of potentially relevant papers retrieved for in-depth review against the inclusion criteria. Discrepancies were resolved by discussion and referral to an additional member of the review team (EK). Full-text articles published in languages other than English were assessed by research trained native speakers working alongside the reviewers to ensure consistency. Figure 1 provides a visual representation of the review’s methodological process, according to the PRISMA framework [29]. Data were independently extracted by two reviewers (SS, KP) using a pre-piloted form in an Excel spreadsheet. Data were extracted on country of origin, year of study and duration; study design and risk of bias assessment; participants’ characteristics; intervention characteristics (including theory base, behavior change technique; modality, delivery agent(s), and training of intervention deliverers, including their professional status; and frequency/duration of exposure), study results and author conclusions, reported analyses, and analysis type. We extracted outcomes in all data forms (e.g., dichotomous, continuous) as reported in the included studies. For dichotomous data, we extracted odds ratios (ORs) with 95% confidence intervals (CIs). For continuous data we extracted mean differences (MD) and 95% confidence intervals.

### 2.4. Data Synthesis

We created summary tables containing characteristics of the studies and their populations, details of the interventions, and outcome measures used. We carried out a narrative synthesis that split the studies into three groups: those that reported linking alcohol consumption and diet in the intervention, those that addressed alcohol consumption and diet separately in the intervention, and those whose primary focus was either alcohol or diet (and included the other). When outcomes were reported using the same or a convertible outcome measure, we pooled the results in a random-effects meta-analysis in RevMan 5.1. Data reported over different time periods were converted to 7 days and the standard errors reported were transformed to standard deviations. The standard deviation (SD) of change scores was rarely reported. If papers included mean change values only within their results, mean change and mean values reported at baseline were used to calculate mean values at follow-up.

### 2.5. Risk of Bias and Appraisal of Methodological Quality

Methodological quality was assessed independently by two reviewers (SS, KP) using the Cochrane Collaboration’s Risk of Bias tool for RCTs [30]. For each item, studies were classified as ‘high’, ‘low’, or ‘unclear’ risk of bias. A ‘summary assessment’ of the risk of bias within each individual study was subsequently derived via Cochrane recommended methodology. A tool adapted from the Cochrane Public Health Review Group’s recommended Effective Public Health Practice Project Quality Assessment Tool for Quantitative Studies was used for non-randomized studies [31]. There were no disagreements in the assessment of methodological quality between the two reviewers and studies were not excluded on the basis of overall quality rating.

## 3. Results

### 3.1. Description of Included Studies

The search provided 10,197 studies for screening, of which 10,005 were excluded during title and abstract screening. A further 182 were excluded during full text screening, leaving eight included studies (Figure 1), as summarized in Table 1. Key study results are provided in Table 2 and intervention content and modality is documented in Supplementary Table S2.

Six studies [32,33,34,35,36,37] reporting four different interventions (U@Uni, Project Fitness, KBS and an unnamed intervention) targeted both diet and alcohol consumption together, and each of them also targeted other health behaviors such as physical activity or smoking. Two of these interventions (KBS, Project Fitness) are explicitly linked dietary and alcohol behaviors in the intervention. A further intervention (HEYMAN) [38] aimed to ‘improve eating habits, activity levels, and overall well-being’, but also contained signposting to resources for reducing alcohol consumption. Another study aimed to reduce cardiovascular disease risk factors; the intervention comprised ‘education about healthy lifestyles’ and alcohol was reported as an outcome measure [39]. One study [38] recruited men aged 18 to 25, and the other studies targeted students.

Theoretical frameworks for interventions predominantly clustered in the domain of health psychology, and spanned Social Cognitive Theory [38], Theory of Planned Behaviour [32,33,35], Self-determination Theory [38], Self-affirmation Theory [32,33], Transtheoretical Model of Change [35], the Behaviour Image Model (BIM) [36,37], Prospect Theory [36], and Message Framing [36]. Two (of 8) studies did not outline any theoretical basis for their intervention [34,39], whilst two interventions were explicitly informed by participatory and formative research [32,33,38]. Three (of six) interventions (Project Fitness, KBS, unnamed intervention) were delivered as a single session [34,35,36,37] whilst the remaining three were delivered across a number of modules [32,33,38,39]. Interventions delivered as a single session still required continued participant engagement post-intervention. Three interventions were exclusively web-based (U@Uni, KBS, unnamed intervention), with specific mechanisms including email prompts to visit the website, video messages delivered by peer coaches, Twitter feeds and Google+ pages and online planners [32,33,34,35]. One further intervention combined a responsive website and a private Facebook discussion group with five other program components including a physical activity tracker, weekly face-to-face sessions, personalized nutrient reports, and ‘dinner discs’ to guide portion sizing (HEYMAN) [38]. There appeared to be a greater degree of personalization/tailoring in this program, with extensive formative research (qualitative focus groups and an online survey) conducted to identify young men’s motivators, barriers and preferences. The final three interventions were delivered wholly face-to-face (Project Fitness, unnamed intervention) [36,37,39].

Face-to-face sessions (including video messages) were delivered by a range of people including degree tutors [39], researchers specializing in education, dietetics and nutrition [38], and ‘fitness specialists’ (trained bachelor’s level research staff) [36]. Personalization (or tailoring) appeared to be a key component of included interventions and formed a central element of most intervention descriptions. Thus, baseline screening was used to ‘script’ intervention content and derive tailored goal setting and individualized sessions. One digital intervention used values that participants described as important to them (e.g., a sense of humor) and hobbies or interests [32,33]. Resultant information formed part of the user’s ‘profile’, which was displayed in the banner at the top of all pages of the intervention website that included the participant’s name, the value that they chose and the reason why it was important to them, in a similar capacity to social media pages such as Twitter, Facebook, and Instagram. One further intervention tested the impact of personalized (versus normative) feedback explicitly [35], whilst, across other interventions, personalization comprised ‘social’ elements, such as group based sessions and team recreational sports [38].

Five studies contained an assessment only control group [32,33,34,35,38], of which one was a waitlist control group [38]. One study provided control participants with ‘standard care’ defined as general health information in the form of leaflets [37]. Two studies did not contain control groups as such: one comprised three active intervention conditions [36], and the other was described as a ‘quasi-experimental study’, but actually provided pre/post data from a single group [39]. All data were continuous, with reported means at follow-up and standard deviations extracted; all studies had comparable follow-up periods (12 weeks to 6 months post-intervention).

### 3.2. Critical Appraisal

A full breakdown of the study critical appraisal is provided in Supplementary Tables S3 and S4. Of six RCTs, three were at low risk of bias [32,33,38], two were classified as being at unclear risk of bias [34,36], and one at high risk of bias due to attrition bias [37]. In the latter study, significantly more students who dropped out received B grades rather than A grades, reported a family alcohol or drug problem and had used marijuana in the past 30 days. The quality of non-randomized studies was classified as weak (*n* = 2), predominantly due to lack of methodological detail reported. For example, Quartiroli et al. [35] recruited students and required them to complete three surveys, then randomised those who had completed the surveys to two intervention groups, and considered those who had answered one or two surveys as the control group (or ‘general sample’).

### 3.3. Effects of Interventions

#### 3.3.1. Interventions Providing Linked Feedback

Two interventions provided linked feedback on alcohol and dietary consumption (KBS, Project Fitness) [35,36,37], and the study results were inconclusive. Linked feedback is defined here as feedback delivered to participants which explicitly linked their current dietary and alcohol behaviors together, or linked any future goal setting together. The majority of participants in Quartiroli et al.’s KBS study (>80%) reported healthy or very healthy lifestyles at baseline in terms of fruit and vegetable and alcohol consumption, and the intervention had little effect on either behaviour after eight weeks. One Project Fitness study [37] reported a small reduction in mean days consuming alcohol (intervention mean 2.41, control 2.77, *p* = 0.00) and mean heavy drinking days per week (intervention 1.74, control 2.03, *p* = 0.00) in the intervention group (Project Fitness consultation) compared to assessment only control after three months, but no impact on amount of alcohol consumed or dietary behaviors. The other Project Fitness study [36] compared the Project Fitness consultation to the completion of a goal-setting contract and log, and to both consultation and contract. It concluded that Project Fitness affected participants’ fruit and vegetable servings and drink driving behaviors (riding with a drinker and drinking whilst driving), but not alcohol consumption over the previous 30 days. However, the consultation and consultation + contract groups were more likely to set goals around drinking post-intervention than the contract only group.

#### 3.3.2. Interventions Targeting Alcohol and Diet Separately 

**Figure 2:**
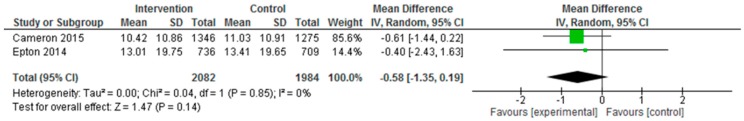
Meta-analysis of volume consumed in units.

**Figure 3:**
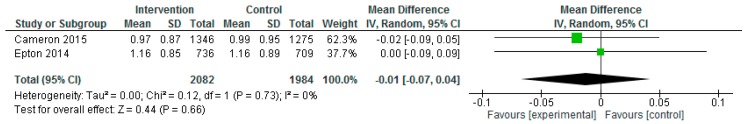
Meta-analysis of number of binge drinking episodes per week.

Two interventions targeted alcohol and dietary consumption separately (U@Uni, unnamed intervention) [32,33,34]. In the two U@Uni trials [32,33], mean consumption of fruit and vegetables per day and alcohol per week were both within recommended limits at baseline. Alcohol consumption data from the U@Uni trials were pooled in two meta-analyses; these were the only studies in this review that reported common or convertible measures of alcohol consumption. The first suggested a possible mean reduction of 0.6 units (95% CI −1.35 to 0.19) of alcohol consumed per week, see Figure 2. The second suggested very little difference in the number of high intensity drinking episodes per week between those who received the intervention and those who didn’t (−0.01 sessions; CI −0.07 to 0.04), see Figure 3. These trials reported conflicting results for changes in fruit and vegetable intake in the intention to treat analysis.

**Figure 4:**
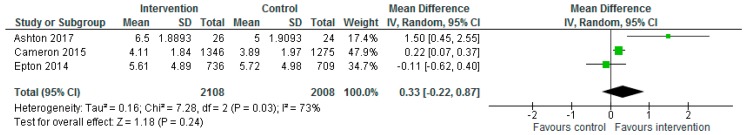
Meta-analysis of daily servings of fruit and vegetables.

According to Kypri et al. [34], participants receiving assessment plus a feedback intervention reported a higher fruit and vegetable consumption after six weeks (33% compliant with guidelines) compared to the minimal contact group (13% compliant), but not compared to the assessment-only group (24% compliant), suggesting possible assessment reactivity. There was no evidence of an impact from this intervention on compliance with alcohol consumption guidelines. The final two interventions (HEYMAN, unnamed intervention) [38,39] were primarily focused on diet and physical activity, but contained a reference to alcohol consumption and reported it as an outcome. Leiva et al. [39] reported that, immediately post-intervention, daily consumption of fruits and vegetables increased to 55%, with 20% of students complying with recommended intake; and that the percentage of at risk drinking decreased from 50% to 38.4%. These changes were not statistically significant. The HEYMAN intervention [38] showed an increase of 1.1 vegetable servings/day (95% CI 0.1 to 2.0) and a larger reduction in percentage of energy dense nutrient poor foods (7.2%, 95% 12.3 to 2.1) in the intervention group compared to control at 3 months. There was no significant change in fruit as reported separately in Table 2. The U@Uni and HEYMAN studies [32,33,38] reported outcomes that could be converted to fruit and vegetable servings per day. The pooled estimate demonstrated that, compared to those in the control condition, people receiving the intervention appeared to increase their daily servings of fruit and vegetables by 0.33 (95% CI −0.22 to 0.87) (see Figure 4). However, this change was not significant.

### 3.4. Physiological Outcomes

We found some evidence of positive changes in a number of physiological measures post-intervention in three (of 8) included studies. Thus, Leiva et al. [39] reported significant reductions in the prevalence of hyperglycaemia (−10.0%; *p* = 0.048) and high blood pressure (−16.7%; *p* = 0.0008), but an increase in High Density Lipoprotein (HDL) cholesterol (pre: 48.7 ± 11.5; post: 53.5 ± 10.4; *p* ≤ 0.0001) among Chilean university students (mean age: 20.7 years). Reduction in prevalence of high blood pressure was particularly significant for women (−15.9%; *p* = 0.005). Further, total cholesterol (pre: 206.1 ± 43.3; post: 185.8 ± 29.9; *p* ≤ 0.0001) and triglycerides (pre: 134.0 ± 69.9; post: 117.0 ± 41.2; *p* = 0.003) only decreased significantly in women. Ashton et al. [38] also demonstrated significant differences favouring the intervention group over the control group at 3-months for plasma total cholesterol (*p* < 0.05, d = 0.60), Low Density Lipoprotein (LDL) cholesterol (*p* < 0.01, d = 0.83), and ratio of total cholesterol-to-HDL cholesterol (*p* < 0.05, d = 0.60). Meanwhile, Cameron et al. [33] identified that the U@Uni intervention had a significant effect on the biochemical marker of alcohol use (fatty acid ethyl esters) at 6-month follow-up, with lower levels of fatty acid ethyl esters observed among participants in the intervention versus the control condition (control: 7.29, SD: 7.85; intervention: 5.00, SD: 4.33, *p* = 0.038).

Four interventions also reported positive changes in additional diet and weight status related outcomes. Leiva et al. [39] reported significant reductions in body weight (pre: 67.7 kg ± 10.8; post: 65.6 kg ± 9.3; *p* ≤ 0.0001), and body mass index (pre: 24.8 ± 3.9; post: 23.9 ± 3.3; *p* ≤ 0.0001), observed post-intervention. Again, this study highlighted gender-specific intervention effects with waist circumference (pre: 78.2 cm ± 10.1; post: 79.2 cm ± 12.0; *p* = 0.0005), which only decreased significantly in women. Ashton et al. [38] identified significant within-group differences in the intervention group for diet quality score, assessed using the Australian Eating Survey FFQ (5.9 95% CI = 3.1, 8.7). This study also found significant differences favouring the intervention group over the control group at 3-months for percentage energy from Energy Dense Nutrient Poor (EDNP) foods (*p* < 0.01, d = 0.73), weight (*p* < 0.05, d = 0.63), percentage weight loss (*p* < 0.05, d = 0.67), waist circumference (*p* < 0.001, d = 0.89), BMI (*p* < 0.01, d = 0.81), and body fat mass (*p* < 0.05, d = 0.67). Finally, for Werch et al. [36], univariate tests showed increases in the consumption of foods containing healthy fats in the past 7 days (e.g., vegetable oil, seeds, nuts, olive oil, or fish) over time (F(1,145), 4.67, *p* = 0.03) post-intervention.

## 4. Discussion

This review reports results from preventative interventions that included both unhealthy eating behavior and alcohol use as the focus or as part of a general intervention in 18–25-year-olds, with most studies conducted among student populations. Pooled estimates demonstrated inconclusive evidence that receiving an intervention resulted in changes to self-reported fruit and vegetable consumption (mean change/daily servings: 0.33; 95% CI −0.22 to 0.87) and alcohol consumption (mean reduction of 0.6 units/week; CI −1.35 to 0.19). There was also no difference in the number of binge drinking episodes per week between intervention and control groups (−0.01 sessions; CI −0.07 to 0.04).

We found some evidence of positive changes in a number of biological and biochemical measures, such as high blood pressure (hypertension) post-intervention in three (of 8) included studies. Our review was not designed to identify studies with these outcomes, this was a secondary aim. As our review highlighted only a small body of research overall, evidence of biological or physiological change is limited. Hazardous and harmful alcohol use and high blood pressure are key risk factors related to premature non-communicable disease (NCD) mortality worldwide [40]; whilst alcohol use/heavy consumption is one of the least intervened risk factors in the management of hypertension at the primary care level [41]. Widespread use of self-reported measures of health behaviors, such as alcohol use and food intake, has been questioned in the academic public health field [42]. Risk of bias assessments used did not include a measure of social desirability bias, and underreporting or misreporting is a major drawback of dietary assessment methods and self-reported alcohol consumption [43,44]. Whilst biomarkers themselves can be subject to error [45], it is possible that bodily or physiological changes are less subject to response/recall bias. Use of biological or physiological markers in future intervention work in this age group may provide stronger evidence for change than self-reported measures alone and should ideally be included as primary outcomes in studies of this nature or future studies. Further, we found evidence of gendered intervention effects within some markers such as waist circumference. Two recent reviews have demonstrated that interventions designed with an understanding of the effect of gender roles, norms, and behaviors on both men’s [46] and women’s health remain limited [47]; whilst an additional ‘umbrella’ review focusing on population-level interventions found gender poorly reported, making it difficult to assess the intended and unintended effects of such policies on women and men [48].

We identified just 8 relevant studies for inclusion in this review. Overall, the quality of the body of evidence we identified was variable. Three studies categorized to be at low risk of bias, focusing on the ‘HEYMAN’ and ‘U@Uni’ interventions, respectively [32,33,38], reported no significant differences in relation to alcohol use. Across included studies, dietary intake seemed to show greater propensity to change than alcohol consumption. Thus, the ‘HEYMAN’ intervention led to significant differences in daily vegetable consumption among Australian young men aged 18–25, whilst results from the U@Uni intervention showed promise in relation to fruit and vegetable intake among UK university students (mean age: 18.76 years) [32,33,38]. It is important to note here that daily servings of fruit and vegetable consumption alone may not capture unhealthy food consumption associated with episodic drinking. For example, findings from other work packages within this project have identified that, whilst young adults may well consume five helpings of fruit and vegetables on most days of the week, they may also eat high calorie/energy dense fried food such as chips, pizza, or fry-ups before bed or the day after drinking alcohol. For linked unhealthy eating and alcohol consumption we need to know the latter rather than the former. Nevertheless, there were notable similarities between these two interventions, which may provide us with ‘take home’ messages for future intervention work in this area. Both interventions outlined an explicit health psychology theoretical basis for intervention content (Social Cognitive Theory and Theory of Reasoned Action and Planned Behaviour, respectively); both comprised distinct program components or modules, some or all of which were digital. In particular, the HEYMAN intervention combined digital elements with face-to-face components, an approach supported by wider literature relating to digital behavior change interventions [49].

There remains a lack of qualitative research exploring the detailed links between alcohol use and eating behaviors in young adults, making it difficult to judge why young adults might be more likely to alter their dietary behaviors than their alcohol use. We can, however, hypothesize several (inter-linked) reasons for this, and extrapolate from related fields in health psychology and applied qualitative health research. First, it may be easier to ‘enhance’ one behavior (i.e., substitute unhealthy food for healthy food) than ‘reduce’ another (alcohol intake). Substitution and reduction are two different behavioral processes, which may warrant different intervention pathways. This may be associated with habit formation where it is much easier to substitute a similar behavior than reduce an entrenched habit, illustrated by smoking cessation research, where a lot of the alternatives to smoking are a substituting behavior [50]. Second, young adults may not view alcohol consumption in the same way as food intake. The pleasure, enjoyment and social norms of drinking alcohol, including the role of masculinity in young men, can be difficult bonds to break [51,52,53,54,55] and young adults may not recognize (or may not want to recognize) alcoholic drinks as calorific; whereas, food intake in this population group is often strongly associated with weight status and appearance [56,57]—combined with the results in a difficult intervention message to navigate.

There are several limitations of our review that should be acknowledged. First, included interventions did not necessarily focus on ‘at risk’ populations. For example, some study populations were relatively ‘healthy’ populations and had low levels of drinking at baseline. Second, the legal drinking age in some countries is 16 meaning that some studies may have been missed by restricting our eligibility criteria to 18 years of age as the lower end of the criteria cut-off. Nevertheless, we thought carefully about our definition of young adulthood within this study. Whilst there are competing definitions of young adulthood (the term ‘young people’ can cover the age range 10–24 years), we chose to focus on 18–25-year-olds as 18-years is the age at which young people in the UK are categorized as an adult by law, and therefore can both legally drink and transition from children to adult services. In addition, this is the age range during which, for some, major life transitions will occur, with the environments of 16–17-year-olds often experientially very different to that of those aged 18 and over. Third, and most importantly, findings from this review offer limited insight into the ability of an intervention to change both alcohol use and unhealthy eating behaviours as: (a) Only a small number of relevant papers were identified; and (b) no outcome measure explicitly linked alcohol use and eating behaviors together. Our extensive literature search included some grey literature such as conference proceedings and theses (through Web of Knowledge and Scopus), which reduced the risk of publication bias by picking up studies that may not have been published in peer reviewed journals. However, it is possible that further unpublished reports might have been revealed with further web searching. We also attempted to contact authors whose papers did not contain SDs or other information useful in the meta-analysis, as well as authors whose papers we were unable to obtain during database searches, with very little success. Further, for some interventions, alcohol use or unhealthy eating behaviors tended not to be the primary focus of the intervention. In this respect, the measures presented are an indicator of the effectiveness of included interventions rather than of the relationship between certain measures of eating behavior (i.e., fruit and vegetable intake) and risky alcohol use. Therefore, this review questions how best to target linked unhealthy eating and alcohol consumption in future intervention work, and what an appropriate outcome measure for such work would be. Our work suggests three potential intervention pathways-linked consumption (such as energy intake from alcohol, total calories consumed across 48 h including a period of intensive or steady alcohol consumption, total calories from alcohol over a specified time period); targeting alcohol use as the primary behavior (whilst acknowledging that alcohol use leads to unhealthy eating practice); or, thirdly, treating alcohol as part of overall diet, thus according both alcohol use and unhealthy eating practices the same level of combined intervention and attention.

## 5. Conclusions

We found limited evidence to suggest that targeted psychosocial interventions focusing on dietary behaviors and risky drinking can improve self-reported behavior in 18–25-year-olds. However, we did identify some evidence of changes in a number of physiological measures post-intervention such as hypertension and waist circumference. Furthermore, we found no intervention content or outcome measure which explicitly linked alcohol use and eating behaviors together (i.e., calories from alcohol consumption, percentage energy intake from alcohol, and changes in diet before, during or after alcohol consumption), and the relationship between these behaviors remains understudied. Of those we did identify, web-based interventions which included a personalized component were common and reported greater propensity for change, particularly in relation to diet. Whilst this review focused on individual-level interventions, it is clear that links between food and alcohol are exploited in marketing based on price (‘Dine in for 2 for £10’) as well as branding (‘https://www.shortlist.com/food-drink/walkers-beer-crisps-max-strong/341587’). Thus, legislative interventions to tackle the price, availability and marketing of unhealthy commodities are endorsed by the World Health Organization, and others, and are likely to be both effective and cost-effective in reducing the harms from these linked behaviors [58]. Nevertheless, individual interventions also have a role to play in motivating and supporting people to change their behavior. The collation of existing evidence, ideally within the form of a systematic review, forms an important part of the development-evaluation-implementation process of intervention development [59]. Findings from this review therefore have important implications for intervention development and point to a need for qualitative research to inform intervention content and appropriate outcome measures targeting linked dietary and alcohol behaviors in this population group.

## Figures and Tables

**Table 1:** Characteristics of included studies.

Reference. Country	Setting	Study Design	RoB/Quality Assessment	Follow Up	Study Aim	Sample Characteristics	Inclusion/Exclusion Criteria	Outcome Measures:	Statistical Methods Used
Ashton et al. (2017) Australia	Region/community wide	Two-arm pilot RCT with waitlist control	Low Risk	3-months post-intervention	To evaluate the feasibility of a targeted healthy lifestyle program for young adult men aged 18–25 years; to estimate the treatment effect of HEYMAN on improving objective physical activity levels (steps/day), diet quality and subjective well-being and other lifestyle, psychological, anthropometric and physiological measures.	*N* = 50 Age range: 1825 Mean age: 22.1 % Single: 80 % Student: 62 % Low income: 48 % High school education or higher: 98	Inclusion: male; aged 18–25; available for assessment sessions; access to a computer, tablet or smartphone with e-mail and Internet facilities. Exclusion: self-reported meeting national recommendations for F&V intakes and/or physical activity; currently participating in an alternative healthy lifestyle program; history of major medical problems (such as heart disease or diabetes that requires insulin injections) and not granted GP approval to participate; reported psychological distress and no GP approval (or associated expert) to participate; diagnosed with an eating disorder; non-English speaking; disability that precluded participation.	Primary: physical activity (pedometer steps/day); diet quality; subjective wellbeing and mental health. Secondary: AUDIT-C; BMI; waist circumference; energy intake (KJ/day); daily servings of fruit and vegetables; proportion of energy from alcohol; proportion of ED-NP foods; MVPA minutes/week. Biomarkers: Fasting Total cholesterol, HDL-Cholesterol, LDL-Cholesterol and Triglycerides (composite measures); Systolic and diastolic blood pressure (composite measures), resting heart rate and augmentation index; salivary cortisol.	Independent *t*-tests and chi-squared (χ2) tests; generalized linear mixed models for intention-to-treat (ITT) populations. Differences of means and 95% confidence intervals (CIs) determined using the mixed models. All health outcomes were included in the model, the predictors included time (treated as categorical with levels baseline and 3 months), treatment group (intervention and control), and an interaction term for time by treatment group. Models adjusted for baseline values of BMI, pedometer steps and proportion of energy from energy-dense, nutrient-poor foods.
Epton et al. (2014) Cameron et al. (2015) UK	University	Two-arm RCT followed by a two-arm Repeat RCT	Low risk	6-months post-intervention	To assess the efficacy and cost-effectiveness of a theory-based online health behaviour intervention targeting health behaviours in new university students (fruit and vegetable intake, physical activity, alcohol consumption and smoking status), in comparison to a measurement-only control.	- Epton et al (2014): *N* = 1445 - Mean age: 18.9 - %Female: 58 • Cameron et al (2015): - *N* = 2621 - Mean age: 18.76 - %Female: 54.1 - %Non-UK students: 57.8	Inclusion: Incoming first year undergraduates Exclusion: NR	Primary: Portions of fruit and vegetables per day; physical activity in the last week; alcohol consumption in the last week (units/week; binge/week); AUDIT; smoking status at 6-month follow up. Secondary: health status; recreational drug use; BMI; health service usage; academic performance; social cognitive variables. Biomarkers: hair sample (3 cm long) liquefied and analysed for biochemical markers of various health behaviours related to alcohol consumption, cigarette smoking, and recreational drug use.	A series of ANCOVAs and logistic regression analyses were used to assess the impact of the intervention on performance of the targeted behaviours at 6-month follow-up, controlling for corresponding baseline scores, sex, age and nationality (i.e., UK or non-UK). For primary outcomes, the Bonferroni correction was used to account for multiple tests. Statistical significance was declared if any of the primary endpoints were significant at 0.0127.
Kypri and McAnally (2005) New Zealand	Student Health Service	Three-arm parallel group RCT	Unclear risk	6-weeks post-intervention	To examine the efficacy of a brief web-based intervention for multiple risk behaviors in a primary care setting for young people.	*N* = 218 Age range: 17–24 Mean age: 20.2 % Female: 49 % European: 75	Inclusion: NR Exclusion: NR	Daily fruit and vegetable consumption; alcohol consumption (age at first drink, alcohol consumption in the past year, largest amount consumed in the last 4 weeks, AUDIT); smoking, physical activity, mental health. * Primary outcome(s) not defined.	Dichotomous variables analysed using Pearson’s Chi-squared test with one degree of freedom for the following pairwise comparisons: A vs. C, A vs. B, and B vs. C (see Supplementary Table S2). Mean peak EBACs and 95% confidence intervals computed for each experimental group; mean differences analysed using analysis of variance.
Leiva et al. (2015) Chile	University	Pre/post	Weak	Immediately post-intervention	To evaluate the effect of a lifestyle-based intervention on reducing cardiovascular risk factors in university students.	*N* = 60 Mean age: - F: 20.7 ± 0.9 years - M: 20.7 ± 1.4 years %Female: 73	Inclusion: Third year students. Exclusion: NR	BMI; physical activity; fruit and vegetable consumption; tobacco use; alcohol consumption. Biomarkers: glucose; total cholesterol (TC); triglyceride (TG); LDL cholesterol; HDL cholesterol; blood pressure. * Primary outcome(s) not defined.	Results presented as mean values with their respective standard deviation (continuous variables). To determine significance between pre and post intervention, t-test was applied for paired samples. For categorical variables, results were presented as prevalence. To determine significant changes in prevalence pre and post-intervention, X2 test was applied.
Quartiroli and Zizzi (2012) USA	University	Pseudo experimental (two-arm)	Weak	8 weeks post-intervention	To pilot test a theory-based, computer-tailored feedback system for improvement of lifestyles among college students at a large, public university.	*N* = 1301 General sample: - N = 303 - Mean age: NR - %White: 84.2 - %Freshman: 67 - %Male: 53.8 - %Residence Halls: 73.3 Intervention sample: - *N* = 62 - Mean age: 19.39 - % White: 93.7 - % Freshman: 58.7 - % Female: 58.7	Inclusion: NR Exclusion: NR	Physical activity (days with moderate physical activity, days with stretching, days with strength activity); daily fruit and vegetable servings; alcohol use (days with at least one drink, number of drinks per day, days with 5+ drinks in a week, number of episodes with 5+ drinks in a month). * Primary outcome(s) not defined.	The impact of intervention was analysed by running a series of 2 (feedback type) × 3 (time) repeated measure ANOVAs, run for each of the dependent variables. In these analyses the independent variables were the assigned group (Normative vs. Personalized) and the time points during the intervention (T1, T2, T3).
Werch et al. (2008) USA	University	Two-arm RCT	High risk	12 weeks post-intervention	To examine the efficacy of a brief, image-based Multiple Behaviour Intervention (MBI) compared against a standard care control for influencing risk behaviors (i.e., alcohol, cigarette, and marijuana consumption and problems) and health-promoting behaviors (i.e., exercise, nutrition, sleep, stress management) as well as health quality of life, among a sample of college students 3 months post-intervention.	N = 303 Age range: 18–21 Mean age: 19.2 %Female: 59.5 %Caucasian: 71.6 %Residence Halls: 44.8	Inclusion: Students aged 18–21 years currently enrolled at the target university and who visited the campus medical services center. Exclusion: NR	Alcohol, cigarette and marijuana consumption (initiation of use, 30-day frequency, 30-day quantity, 30-day heavy use); 18-item measure of alcohol and drug problems; physical activity (initiation of exercise, 30-day vigorous physical activity, 30-day moderate physical activity, 7-day strenuous exercise, 7-day moderate exercise); nutrition habits (past 30-day servings of fruit and vegetables, number of times eating healthy carbohydrates and fats); sleep habits; self-reported health status. * Primary outcome(s) not defined.	Baseline measures were compared across treatment group using chi-square tests for categorical variables and independent sample *t*-tests for continuous variables. Repeated measures MANOVAs and ANOVAs were used to test intervention effects over time. Repeated-measures MANOVAs were performed to more efficiently address the multiple health behaviours targeted by the intervention. Effect sizes were calculated based on mean pre–post change in the treatment group minus the mean pre–post change in the control group, divided by the pooled pre-test standard deviation.
Werch et al. (2007) USA	University	Three-arm RCT	High risk	1-month post-intervention	To examine the effects of brief image-based interventions, including a multiple behavior health contract, a one-on-one tailored consultation, and a combined consultation plus contract intervention, for impacting multiple health behaviors of students in a university health clinic.	*N* = 155 Mean age: 19 % Female: 66 % Caucasian: 52 % Live off-campus: 56	Inclusion: Students currently enrolled at the target university. Exclusion: NR	Alcohol, cigarette and marijuana consumption (length of use, 30-day frequency, 30-day quantity); physical activity (30-day vigorous physical activity, 30-day moderate physical activity, 7-day strenuous exercise, 7-day moderate exercise); nutrition habits (past 7-day servings of fruit and vegetables, number of times eating good carbohydrates and fats); sleep habits; self-reported health status. * Primary outcome(s) not defined.	Baseline measures were compared across treatment group using chi-square tests for categorical data and ANOVA tests for continuous scores. Both ANOVAs and repeated-measures MANOVAs were used to test intervention effects over time, first, on behaviour measures and, second, on image and belief measures. Repeated-measures MANOVAs were performed to more efficiently address the multiple health behaviours targeted by the intervention, and because the dependent variables were not perfectly correlated.

**Table 2:** Key study results against behavioural outcome measures.

Reference:	Results:
Ashton et al. (2017)	No significant differences between groups observed for alcohol consumption (0.7, 95% CI = −0.3, 1.8, *p* = 0.181, d = 0.36) or diet quality score (3.6, 95% CI = −0.4, 7.6, *p* = 0.081, d = 0.48). Significant within-group differences evident in the intervention group for diet quality score (5.9 95% CI = 3.1, 8.7). Significant differences favouring the intervention group at 3-months were observed for daily vegetable servings (*p* < 0.05, d = 0.62), percentage energy from EDNP foods (*p* < 0.01, d = 0.73), weight (*p* < 0.05, d = 0.63), percentage weight loss (*p* < 0.05, d = 0.67), waist circumference (*p* < 0.001, d = 0.89), BMI (*p* < 0.01, d = 0.81), body fat mass (*p* < 0.05, d = 0.67), plasma total cholesterol (*p* < 0.05, d = 0.60), LDL cholesterol (*p* < 0.01, d = 0.83) and ratio of total cholesterol-to-HDL cholesterol (*p* < 0.05, d = 0.60).
Epton et al. (2014) Cameron et al. (2015)	Epton et al.: At 6-month follow-up, fruit and vegetable intake and alcohol consumption did not differ significantly between the two arms (F&V portions: Control: mean: 5.72, SD: 4.98; Intervention: mean: 5.61, SD: 4.89, *p* = 0.708, d = −0.02; mean alcohol units in past 7 days: control: 13.41, SD: 19.65, intervention: 13.01, SD: 19.75, *p* = 0.737, d = 0.02; mean no. binge drinking days in past 7 days: control: 1.16, SD: 0.89, intervention: 1.16, SD: 0.85, *p* = 0.973, d = 0.00). Cameron et al.: No significant differences between the intervention and control conditions on the primary outcomes at 6-month follow-up, although the effect of the intervention on fruit and vegetable intake approached significance (fruit and vegetable mean portions per day (control: 3.89, SD: 1.97; intervention: 4.11, SD: 1.84; *p* = 0.024); mean alcohol units in the past 7 days (control: 11.03, SD: 10.91; intervention: 10.42, SD: 10.86, *p* = 0.277). Repeating the primary analyses without data imputation produced consistent results. No significant differences between the intervention and control conditions at 6-month follow up in relation to no. days binge drinking in past 7 days (control: 0.99, SD: 0.95; intervention: 0.97, SD: 0.87, *p* = 0.674). Effect sizes found in the repeat trial were comparable to those found in the original trial for fruit and vegetable intake, Q(1) = 2.93, *p* = 0.087, and mean alcohol units in past 7 days, Q(1) = 0.25, *p* = 0.619. A marginally larger effect size was found for fruit and vegetable intake in the repeat trial (d = 0.12) than in the original trial (d = −0.02). Per-protocol analyses demonstrated intervention participants reported consuming significantly more portions of fruit and vegetables, F(1, 1068) = 7.19, *p* = 0.007, than those in the control condition (mean = 4.23, 3.89; SD = 0.11 and 0.07, respectively). Like the primary analyses, there was no significant effect of the intervention on alcohol units consumed, F(1, 1030) = 1.30, *p* = 0.254. The intervention had a significant effect on the biochemical marker of alcohol use (fatty acid ethyl esters) at 6-month follow-up, with lower levels of alcohol use observed among participants in the intervention versus control condition (control: 7.29, SD: 7.85; intervention: 5.00, SD: 4.33, *p* = 0.038).
Kypri and McAnally (2005)	Fruit and vegetable consumption: Group A had significantly greater compliance with recommendations than group C. Differences between A and B, and B vs. C were non-significant (*p* value: A vs. C: 0.02; A vs. B: 0.44; B vs. C:0.08). Alcohol consumption: None of the groups differed significantly in their compliance with recommended limits for episodic alcohol consumption (based on binge criteria) (*p* value: A vs. C: 0.84; A vs. B: 0.44; B vs. C: 0.70). The mean (95% confidence interval) peak EBACs in groups A, B, and C were 0.11 (0.08, 0.14), 0.12 (0.09, 0.15), and 0.13 (0.10, 0.15), F = 0.208, *p* = 0.813.
Leiva et al. (2015)	Significant reductions in the prevalence of hyperglycaemia (−10.0%; *p* =0.048) and high blood pressure (−16.7%; *p* = 0.0008) observed post-intervention. Reduction in prevalence of high blood pressure was particularly significant for women (−15.9%; *p* = 0.005). Significant reductions in body weight (pre: 67.7 ± 10.8; post: 65.6 ± 9.3; *p* ≤ 0.0001), body mass index (pre: 24.8 ± 3.9; post: 23.9 ± 3.3; *p* ≤ 0.0001), blood pressure (pre: 103.2 ± 11.2; post: 98.0 ± 8.3; *p* ≤ 0.0001), and an increase in HDL cholesterol (pre: 48.7 ± 11.5; post: 53.5 ± 10.4; *p* ≤ 0.0001) observed post-intervention. Waist circumference (pre: 78.2 ± 10.1; post: 79.2 ± 12.0; *p* = 0.0005), total cholesterol (pre: 206.1 ± 43.3; post: 185.8 ± 29.9; *p* ≤ 0.0001) and triglycerides (pre: 134.0 ± 69.9; post: 117.0 ± 41.2; *p* = 0.003) only decreased significantly in women. Other health markers such as baseline glycaemia, PAD and cholesterol LDL, were unchanged in the study population. Post-intervention, there was no change in the percentage of students who drank alcohol, but the amount of drinks they declared decreased to 38.4% drinkers at risk (from 50.0% pre-intervention; *p* = 0.201). Post-intervention, the daily consumption of fruits and vegetables increased to 55%, with 20% of students declaring that they consume 5+ servings daily, thus complying with the recommendations. However, these changes were not significant.
Quartiroli and Zizzi (2012)	Fruit and vegetable consumption: No significant main effects or interactions were found over time for fruit and vegetable intake (Time 1: mean servings: 7.40, SD: 3.396; Time 2: mean servings: 7.39, SD: 3.423; Time 3: mean servings: 6.98, SD: 3.257). Alcohol consumption: Days w/1 drink: Time 1: mean: 0.93, SD: 1.260; Time 2: mean: 1.08, SD: 1.297; Time 3: mean: 1.15, SD: 1.365. Drinks per day: Time 1: mean: 0.70, SD: 1.094; Time 2: mean: 0.93, SD: 1.436; Time 3: mean: 1.00, SD: 1.0547. Binge/Week: Time 1: mean: 0.60, SD: 1.045; Time 2: mean: 0.61, SD: 1.061; Time 3: mean: 0.69, SD: 1.288 Binge/Month: Time 1: mean: 0.83, SD: 1.044; Time 2: mean: 0.92, SD: 1.076; Time 3: mean: 0.76, SD: 0.862 Alcohol use over time: The interaction of the two variables (days w/1 drink and drinks per day) suggested a small, significant increase in drinks per day [F(2,120)=3.53, *p* = 0.03, ES = 0.058, Obs.Pow = 0.647]. Individuals in the normative feedback group showed a slightly larger change in their average of drinks per day than the personalized feedback group (Personalized −0.14; Normative 0.50). Change in drinking habits was also in the opposite direction for the individuals in the personalized feedback group. Overall, there was not a significant main effect for time (*p* = 0.238) or for group (*p* = 0.527).
Werch et al. (2008)	Post intervention, univariate tests for alcohol behaviours found that students exposed to the brief intervention drank alcohol less frequently (intervention: M=2.41, SE=0.12; control: M = 2.77, SE = 0.12; D = 0.27, *p* = 0.00), drank heavily less frequently (intervention: M = 1.74, SE = 0.10; control: M = 2.03, SE = 0.10; D = 0.29, *p* = 0.00) and drove after drinking less frequently (intervention: M = 0.50, SE = 0.11; control: M = 0.71, SE = 0.10; D = 0.23, *p* = 0.02). No omnibus treatment by time interactions were found for nutrition behaviours (fruits/vegetables: intervention: M = 4.31, SE = 0.17; control: M = 3.73, SE = 0.16, D = 0.20; good carbohydrate: intervention: M = 5.46, SE = 0.23; control: M = 4.76, SD = 0.23, D= 0.23; good fats: intervention: M = 4.34, SD = 0.21; control: M = 3.59, SE = 0.21, D = 0.22). Omnibus treatment by time multivariate analysis of variance interactions were significant for alcohol consumption behaviours groupings (F(6, 261) = 2.73, *p* = 0.01). Small positive effects were found for increases on all three nutrition behaviours (F = 1.33, df = 3, 279, *p* = 0.27), even though overall MANOVA tests were not significant.
Werch et al. (2007)	Omnibus repeated-measures MANOVAs were significant for drinking driving behaviours (F(2,136), 4.43, *p* = 0.01) and nutrition habits, inclusive of fruit and vegetable consumption (F(3,143), 5.37, *p* = 0.00), with improvements on each of these behaviours across time. No differences were seen over time on alcohol consumption measures (F(3,142), 0.48, *p* = 0.69); univariate analyses showed decreases in the frequency of riding with a drunk driver (F(1,145), 9.63, *p* = 0.01), and a near significant decrease in driving while drunk (F(1,137), 3.64, *p* = 0.06). Univariate tests also showed increases in the consumption of foods containing healthy fats in the past 7 days (e.g., vegetable oil, seeds, nuts, olive oil, or fish) over time (F(1,145), 4.67, *p* = 0.03).

## Supplementary Materials

The following are available online at http://www.mdpi.com/2072-6643/10/10/1538/s1, Table S1: Database-specific Search Strategies, Table S2: Intervention Content and Delivery, Table S3: Risk of bias assessment for RCTs, Table S4: EPHPP quality appraisal for non-randomised or experimental studies.
