# Supplementary material for: Non-Pharmacological Interventions to Reduce Unhealthy Eating and Risky Drinking in Young Adults Aged 18–25 Years: A Systematic Review and Meta-Analysis
Source: Nutrients. 2018 Oct 18;10(10):1538. doi: 10.3390/nu10101538 (PMC6213108; doi:10.3390/nu10101538)
Supplement: Supplementary file 1 [file nutrients-10-01538-s001.zip › nutrients-364264-sup/Supplementary Table S1. Database-specific Search Strategies.docx]

**Supplementary Table S1.** Database-specific Search Strategies

### MEDLINE/epub/Central

1. (multi* adj2 health* adj2 behavio?r*).ti,ab.

2. (multi* adj2 unhealth* adj2 behavio?r*).ti,ab.

3. (multi* adj2 risk* adj2 behavio?r*).ti,ab.

4. (co?variat* adj5 behavio?r*).ti,ab.

5. (cluster* adj2 health* adj2 behavio?r*).ti,ab.

6. (cluster* adj2 unhealth* adj2 behavio?r*).ti,ab.

7. (cluster* adj2 risk* adj2 behavio?r*).ti,ab.

8. (risk-taking adj2 behavio?r*).ti,ab.

9. risk-taking/

10. or/1-9

11. exp Alcohol Drinking/

12. exp alcoholic beverages/

13. alcoholic intoxication/

14. (alcohol* adj2 (abuse* or misuse* or use* or consum* or drink* or excess* or problem* or risk*)).mp.

15. alcohol*.mp.

16. ((binge or problem* or risk* or excess*) adj2 drink*).mp.

17. ((hazardous or unsafe or unhealthy) adj2 drink*).mp.

18. drunk*.mp.

19. (intoxicat* adj4 (drink* or alcohol*)).mp.

20. (wine or beer or spirits).mp.

21. or/11-20

22. exp overweight/

23. exp overnutrition/ or hyperphagia/

24. exp "Body Weights and Measures"/

25. Food preferences/

26. *Feeding Behavior/ or exp Food habits/

27. Energy Intake/

28. fast foods/ or carbonated beverages/

29. (obes* or over?weight or over?nutrition).ti,ab.

30. (excess adj2 weight).ti,ab.

31. ((eat* or food* or feed*) adj2 (behavio?r* or excessive* or choice? or pattern? or habit? or preference?)).ti,ab.

32. (body adj2 (mass or size or weight)).ti,ab.

33. (diet* or nutrition).ti,ab.

34. over?eat*.mp.

35. (under?weight or under?nutrition or under?eat).ti,ab.

36. (unhealth* adj2 (diet* or eating or food*)).mp.

37. ((vegetable* or fruit) adj2 (eat* or intake or consum* or portion* or serving? or frequenc* or number? or preference? or choice*)).mp.

38. (((junk or fast or unhealthy or choice? or processed) adj2 food*) or fastfood).mp.

39. (calorie-dense adj2 (food? or beverage? or drink?)).mp.

40. (convenien* adj2 (food* or meal*)).mp.

41. (excess* adj2 (fat* or salt* or sugar*)).mp.

42. (energy adj1 intake).mp.

43. (poor adj2 diet).mp.

44. snack*.mp.

45. (((fizzy or sugary) adj2 drink*) or soda or coca-cola or coke or cola or pop).mp.

46. (take?away or take?out or carry?out).mp.

47. (((frozen or ready or TV or television) adj2 meal?) or ((TV or television) adj2 dinner?)).mp.

48. ((portion or serving) adj2 size?).mp.

49. or/22-48

50. Young Adult/

51. (young adj2 (adult? or person?)).mp.

52. ((college* or university) adj2 student?).mp.

53. late-teen*.mp.

54. early-adult*.mp.

55. (adolescen* or youth* or undergraduate* or freshmen or fresher? or teen* or student?).mp.

56. or/50-55

57. intervention?.mp.

58. (weightloss or (weight adj2 (reduc* or loss))).mp.

59. ((decreas* or reduc*) adj2 (alcohol* or drink*)).mp.

60. ((improve* or health*) adj2 (diet* or food? or choice?)).mp.

61. Health Promotion/

62. Health behavior/

63. Secondary prevention/

64. *risk reduction behavior/

65. (health adj1 (promot* or protect*)).mp.

66. ((modif* or chang*) adj5 (behavio?r* or habit*)).mp.

67. (early adj2 therap*).mp.

68. prevent*.mp.

69. (person* adj2 feedback).mp.

70. or/57-69

71. ((10 and 21) or (10 and 49) or (21 and 49)) and 56 and 70

72. adult/ not (adult/ and (young adult/ or adolescent/))

73. 71 not 72

### Psycinfo

1. (multi* adj2 health* adj2 behavio?r*).ti,ab.

2. (multi* adj2 unhealth* adj2 behavio?r*).ti,ab.

3. (multi* adj2 risk* adj2 behavio?r*).ti,ab.

4. (co?variat* adj5 behavio?r*).ti,ab.

5. (cluster* adj2 health* adj2 behavio?r*).ti,ab.

6. (cluster* adj2 unhealth* adj2 behavio?r*).ti,ab.

7. (cluster* adj2 risk* adj2 behavio?r*).ti,ab.

8. (risk-taking adj2 behavio?r*).ti,ab.

9. risk taking/

10. or/1-9

11. exp Alcohol Drinking Patterns/

12. alcohol drinking attitudes/

13. exp alcoholic beverages/

14. binge drinking/

15. underage drinking/

16. (alcohol* adj2 (abuse* or misuse* or use* or consum* or drink* or excess* or problem* or risk*)).mp.

17. alcohol*.mp.

18. ((binge or problem* or risk* or excess*) adj2 drink*).mp.

19. ((hazardous or unsafe or unhealthy) adj2 drink*).mp.

20. drunk*.mp.

21. (intoxicat* adj4 (drink* or alcohol*)).mp.

22. (wine or beer or spirits).mp.

23. or/11-22

24. exp overweight/

25. eating behavior/

26. exp body weight/ or body mass index/

27. Food preferences/

28. fast food/ or binge eating/ or dietary restraint/

29. (obes* or over?weight or over?nutrition).ti,ab.

30. (excess adj2 weight).ti,ab.

31. ((eat* or food* or feed*) adj2 (behavio?r* or excessive* or choice? or pattern? or habit? or preference?)).ti,ab.

32. (body adj2 (mass or size or weight)).ti,ab.

33. (diet* or nutrition).ti,ab.

34. over?eat*.mp.

35. (under?weight or under?nutrition or under?eat).ti,ab.

36. (unhealth$ adj2 (diet$ or eating or food*)).mp.

37. ((vegetable$ or fruit) adj2 (eat* or intake or consum* or portion* or serving? or frequenc* or number? or preference? or choice*)).mp.

38. (((junk or fast or unhealthy or choice? or processed) adj2 food*) or fastfood).mp.

39. (calorie-dense adj2 (food? or beverage? or drink?)).mp.

40. (convenien* adj2 (food* or meal*)).mp.

41. (excess* adj2 (fat* or salt* or sugar*)).mp.

42. (energy adj1 intake).mp.

43. (poor adj2 diet).mp.

44. snack*.mp.

45. (((fizzy or sugary) adj2 drink*) or soda or coca-cola or coke or cola or pop).mp.

46. (take?away or take?out or carry?out).mp.

47. (((frozen or ready or TV or television) adj2 meal?) or ((TV or television) adj2 dinner?)).mp.

48. ((portion or serving) adj2 size?).mp.

49. or/24-48

50. exp college students/

51. adolescent development/ or adult development/

52. (young adj2 (adult? or person?)).mp.

53. ((college* or universit*) adj2 student?).mp.

54. late-teen*.mp.

55. early-adult*.mp.

56. (adolescen* or youth* or undergraduate* or freshmen or fresher? or teen* or student?).mp.

57. or/50-56

58. intervention?.mp.

59. (weightloss or (weight adj2 (reduc* or loss))).mp.

60. ((decreas* or reduc*) adj2 (alcohol* or drink*)).mp.

61. ((improve* or health*) adj2 (diet* or food? or choice?)).mp.

62. Health Promotion/

63. Health behavior/

64. risk management/

65. (health adj1 (promot* or protect*)).mp.

66. ((modify* or chang*) adj5 (behavio?r* or habit*)).mp.

67. (early adj2 therap*).mp.

68. prevent*.mp.

69. (person* adj2 feedback).mp.

70. or/58-69

71. ((10 and 23) or (10 and 49) or (23 and 49)) and 57 and 70

### CINAHL

S1 ((multi* w2 health*) w2 behavior*) OR ((multi* w2 unhealth*) w2 behavior*) OR ((multi* w2 risk*) w2 behavior*)

S2 ((cluster* w2 health*) w2 behaviour*) OR ((cluster* w2 unhealth*) w2 behaviour*) OR ((cluster* w2 risk*) w2 behaviour*)

S3 covariat* w5 behavior* OR risk-taking w2 behavior*

S4 (MH "Risk Taking Behavior")

S5 S1 OR S2 OR S3 OR S4

S6 (MH "Drinking Behavior+") OR (MH "Alcoholic Beverages") OR (MH "Alcoholic Intoxication")

S7 (alcohol* n2 (abuse* or misuse* or use* or consum* or drink* or excess* or problem* or risk*))

S8 (alcohol* or drunk*) OR (((binge or problem* or risk* or excess*) w2 drink*) ) OR ( ((hazardous or unsafe or unhealthy) w2 drink*) ) OR ( (intoxicat* n4 (drink* or alcohol*)) ) OR ( wine or beer or spirits )

S9 S6 OR S7 OR S8

S10 (MH "Eating Behavior+") OR (MH "Body Mass Index") OR (MH "Body Weight+") OR (MH "Obesity+") OR (MH "Hyperphagia") OR (MH "Fast Foods") OR (MH "Carbonated Beverages")

S11 (obes * or overweight or overnutrition) OR excess* w2 weight OR (((eat * or food * or feed *) n2 (behaviour * or excessive * or choice * or pattern * or habit * or preference *)))

S12 ( (body w2 (mass or size or weight)) ) OR ( diet * or nutrition or overeat* or over-eat * ) OR ( underweight or undernutrition or undereat ) OR ( (unhealth * w2 (diet * or eating or food *)) ) OR ( ((vegetable * or fruit) n2 (eat * or intake or consum* or portion* or serving* or frequenc * or number * or preference * or choice *)) ) OR ( (((junk or fast or unhealthy or choice * or processed) w2 food *) or fastfood) )

S13 ((calorie-dense w2 (food * or beverage * or drink *))) OR ((convenien * w2 (food * or meal*))) OR ( (excess * w2 (fat * or salt * or sugar *)) ) OR (energy w1 intake) OR (poor w2 diet) OR snack *

S14 ((((fizzy or sugary) w2 drink *) or soda or coca-cola or coke or cola or pop)) OR (takeaway or takeout or carryout) OR ((((frozen or ready or TV or television) w2 meal*) or ((TV or television) w2 dinner *))) OR (((portion or serving) adj2 size *))

S15 S10 OR S11 OR S12 OR S13 OR S14

S16 (MH "Young Adult") OR (MH "Students, College") OR (MH "High School Graduates")

S17 ((young w2 (adult* or person*))) OR (((college* or university) n2 student*)) OR (late-teen* or early-adult*) OR (adolescen* or youth* or undergraduate* or freshmen or fresher* or teen* or student*)

S18 S16 OR S17

S19 (MH "Health Promotion") OR (MH "Health Behavior") OR (MH "Preventive Health Care")

S20 ( intervention* or prevent* ) OR ( (weightloss or (weight n2 (reduc* or loss))) ) OR ( ((decreas* or reduc*) n2 (alcohol* or drink*)) ) OR ( ((improve* or health*) w2 (diet* or food* or choice*)) ) OR ( (health n1 (promot* or protect*)) ) OR ( ((modif* or chang*) n5 (behavior* or habit*)) ) OR (early w2 therap*) OR (person* w2 feedback)

S21 S19 OR S20

S22 S5 and (s9 or s15) and s18 and s21

### ERIC via Ebsco

S1 (((multi* w2 health*) w2 behavior*) OR ((multi* w2 unhealth*) w2 behavior*) OR ((multi* w2 risk*) w2 behavior*)) OR (((cluster* w2 health*) w2 behaviour*) OR ((cluster* w2 unhealth*) w2 behaviour*) OR ((cluster* w2 risk*) w2 behaviour*)) OR ((covariat* w5 behavior*) OR (risk-taking w2 behavior*))

S2 ((alcohol* n2 (abuse* or misuse* or use* or consum* or drink* or excess* or problem* or risk*))) OR ( ( alcohol* or drunk* ) OR ( ((binge or problem* or risk* or excess*) w2 drink*) ) OR ( ((hazardous or unsafe or unhealthy) w2 drink*) ) OR ( (intoxicat* n4 (drink* or alcohol*)) ) OR ( wine or beer or spirits ) )

S3 DE "Alcohol Abuse"

S4 S2 OR S3

S5 ( ( obes* or overweight or overnutrition ) OR excess* w2 weight OR ( ((eat* or food* or feed*) n2 (behavior* or excessive* or choice* or pattern* or habit* or preference*)) ) ) OR ( ( (body w2 (mass or size or weight)) ) OR ( diet* or nutrition or overeat* or over-eat* ) OR ( underweight or undernutrition or undereat ) OR ( (unhealth* w2 (diet* or eating or food*)) ) OR ( ((vegetable* or fruit) n2 (eat* or intake or consum* or portion* or serving* or frequenc* or number* or preference* or choice*)) ) OR ( (((junk or fast or unhealthy or choice* or processed) w2 food*) or fastfood) ) ) OR ( ( (calorie-dense w2 (food* or beverage* or drink*)) ) OR ( (convenien* w2 (food* or meal*)) ) OR ( (excess* w2 (fat* or salt* or sugar*)) ) OR (energy w1 intake) OR (poor w2 diet) OR snack* ) OR ( ( (((fizzy or sugary) w2 drink*) or soda or coca-cola or coke or cola or pop) ) OR ( takeaway or takeout or carryout ) OR ( (((frozen or ready or TV or television) w2 meal*) or ((TV or television) w2 dinner*)) ) OR ( ((portion or serving) adj2 size*) ) )

S6 (DE "Body Weight" OR DE "Obesity") OR (DE "Eating Habits")

S7 S5 OR S6

S8 (((young w2 (adult* or person*))) OR (((college* or university) n2 student*)) OR (late-teen* or early-adult*) OR (adolescen* or youth* or undergraduate* or freshmen or fresher* or teen* or student*))

S9 ((DE "Young Adults") OR (DE "College Bound Students")) AND (DE "College Students" OR DE "College Freshmen" OR DE "College Seniors" OR DE "College Transfer Students" OR DE "First Generation College Students" OR DE "Graduate Students" OR DE "In State Students" OR DE "On Campus Students" OR DE "Out of State Students" OR DE "Preservice Teachers" OR DE "Two Year College Students" OR DE "Undergraduate Students" OR DE "College Graduates" OR DE "Late Adolescents")

S10 S8 OR S9

S11 ( ( intervention* or prevent* ) OR ( (weightloss or (weight n2 (reduc* or loss))) ) OR ( ((decreas* or reduc*) n2 (alcohol* or drink*)) ) OR ( ((improve* or health*) w2 (diet* or food* or choice*)) ) OR ( (health n1 (promot* or protect*)) ) OR ( ((modif* or chang*) n5 (behavior* or habit*)) ) OR (early w2 therap*) OR (person* w2 feedback) )

S12 (DE "Health Promotion" OR DE "Health Behavior") OR (DE "Prevention")

S13 S11 OR S12

S14 ((S1 and S4) or (S1 and S7) or (S4 and S7)) and S10 and S13

### Web of Science core collection

# 1 TOPIC: (multi* near/2 (health* or unhealth* or risk*) near/2 behavior*) OR TOPIC: (cluster* near/2 (health* or unhealth* or risk*) near/2 behavior*) OR TOPIC: (covariat* near/5 behavior*) OR TOPIC: (risk-taking near/2 behavior*)

# 2 TOPIC: ((alcohol* near/2 (abuse* or misuse* or use* or consum* or drink* or excess* or problem* or risk*))) OR TOPIC: (alcohol* or drunk* or wine or beer or spirits) OR TOPIC: (((binge or problem* or risk* or excess*) near/2 drink*)) OR TOPIC: ((intoxicat* near/4 (drink* or alcohol*)))

# 3 TOPIC: (obes* or overweight or overnutrition or over-nutrition or diet* or nutrition or overeat* or over-eat* or underweight or undernutrition or undereat* or under-eat* or snack*) OR TOPIC: ((excess near/2 weight)) OR TOPIC: (((eat* or food* or feed*) near/2 (behavior* or excessive* or choice* or pattern* or habit* or preference*))) OR TOPIC: ((body near/2 (mass or size or weight))) OR TOPIC: ((unhealth* near/2 (diet* or eating or food*))) OR TOPIC: (((vegetable* or fruit) near/2 (eat* or intake or consum* or portion* or serving* or frequenc* or number* or preference* or choice*))) OR TOPIC: ((((junk or fast or unhealthy or choice* or processed) near/2 food*) or fastfood)) OR TOPIC: ((calorie-dense near/2 (food* or beverage* or drink*))) OR TOPIC: ((convenien* near/2 (food* or meal*))) OR TOPIC: ((excess* near/2 (fat* or salt* or sugar*))) OR TOPIC: ((energy near/1 intake)) OR TOPIC: ((poor near/2 diet)) OR TOPIC: ((((fizzy or sugary) near/2 drink*) or soda or coca-cola or coke or cola or pop)) OR TOPIC: ((takeaway or take-away or takeout or take-out or carryout or carry-out)) OR TOPIC: ((((frozen or ready or TV or television) near/2 meal*) or ((TV or television) near/2 dinner*))) OR TOPIC: (((portion or serving) near/2 size*))

# 4 TOPIC: ((young near/2 (adult* or person*))) OR TOPIC: (((college* or university) near/2 student*)) OR TOPIC: (late-teen*) OR TOPIC: (early-adult*) OR TOPIC: ((adolescen* or youth* or undergraduate* or freshmen or fresher* or teen* or student*))

# 5 TOPIC: (intervention* or prevent*) OR TOPIC: ((weightloss or (weight near/2 (reduc* or loss)))) OR TOPIC: (((decreas* or reduc*) near/2 (alcohol* or drink*))) OR TOPIC: (((improve* or health*) near/2 (diet* or food* or choice*))) OR TOPIC: ((health near/1 (promot* or protect*))) OR TOPIC: (((modif* or chang*) near/5 (behavior* or habit*))) OR TOPIC: ((early near/2 therap*)) OR TOPIC: ((person* near/2 feedback))

# 6 ((#1 and #2) or (#1 and #3) or (#2 and #3)) and #4 and #5

### ASSIA

S1 SU.EXACT("Risk behaviour") OR ((multi* near/2 (health* or unhealth* or risk*) near/2 behavior*) OR (cluster* near/2 (health* or unhealth* or risk*) near/2 behavior*) OR (covariat* near/5 behavior*) OR (risk-taking near/2 behavior*))

S2 SU.EXACT("Alcoholic beverages" OR "Alcoholic soft drinks" OR "Banana beer" OR "Beer" OR "Designer drinks" OR "Gin" OR "Martinis" OR "Spirits" OR "Wine") OR (((alcohol* near/2 (abuse* or misuse* or use* or consum* or drink* or excess* or problem* or risk*))) OR (alcohol* or drunk* or wine or beer or spirits) OR (((binge or problem* or risk* or excess*) near/2 drink*)) OR ((intoxicat* near/4 (drink* or alcohol*)))) OR SU.EXACT("Alcohol abuse" OR "Alcohol consumption" OR "Alcoholism" OR "Bars" OR "Binge drinking" OR "Familial alcoholism" OR "Hangovers" OR "Heavy drinking" OR "Moderate drinking" OR "Problem drinking" OR "Social drinking" OR "Solitary drinking" OR "Temperance")

S3 SU.EXACT("Obesity") OR SU.EXACT("Fast food") OR SU.EXACT("Hyperphagia") OR SU.EXACT("Underweight") OR SU.EXACT("Food habits") OR ( (obes* or overweight or overnutrition or over-nutrition or diet* or nutrition or overeat* or over-eat* or underweight or undernutrition or undereat* or under-eat* or snack*) OR ((excess near/2 weight)) OR (((eat* or food* or feed*) near/2 (behavior* or excessive* or choice* or pattern* or habit* or preference*))) OR ((body near/2 (mass or size or weight))) OR ((unhealth* near/2 (diet* or eating or food*))) OR (((vegetable* or fruit) near/2 (eat* or intake or consum* or portion* or serving* or frequenc* or number* or preference* or choice*))) OR ((((junk or fast or unhealthy or choice* or processed) near/2 food*) or fastfood)) OR ((calorie-dense near/2 (food* or beverage* or drink*))) OR ((convenien* near/2 (food* or meal*))) OR ((excess* near/2 (fat* or salt* or sugar*))) OR ((energy near/1 intake)) OR ((poor near/2 diet)) OR ((((fizzy or sugary) near/2 drink*) or soda or coca-cola or coke or cola or pop)) OR ((takeaway or take-away or takeout or take-out or carryout or carry-out)) OR ((((frozen or ready or TV or television) near/2 meal*) or ((TV or television) near/2 dinner*))) OR (((portion or serving) near/2 size*)))

S4 SU.EXACT("Autistic young adults" OR "Deaf young adults" OR "Disabled young adults" OR "Emotionally disturbed young adults" OR "Hearing impaired young adults" OR "Hyperactive learning disabled young adults" OR "Learning disabled young adults" OR "Mentally ill young adults" OR "Sick young adults" OR "Special needs young adults" OR "Terminally ill young adults" OR "Young adults") OR (SU.EXACT("Undergraduate students") OR SU.EXACT("Postgraduate students")) OR SU.EXACT("Young adulthood") OR (((young near/2 (adult* or person*))) OR (((college* or university) near/2 student*)) OR (late-teen*) OR (early-adult*) OR ((adolescen* or youth* or undergraduate* or freshmen or fresher* or teen* or student*)))

S5 SU.EXACT("Health promotion") OR SU.EXACT("Health behaviour") OR (SU.EXACT("Preventive programmes") OR SU.EXACT("Preventive health care")) OR ((intervention* or prevent*) OR ((weightloss or (weight near/2 (reduc* or loss)))) OR (((decreas* or reduc*) near/2 (alcohol* or drink*))) OR (((improve* or health*) near/2 (diet* or food* or choice*))) OR ((health near/1 (promot* or protect*))) OR (((modif* or chang*) near/5 (behavior* or habit*))) OR ((early near/2 therap*)) OR ((person* near/2 feedback)))

S6 s1 and s2

S7 s1 and s3

S8 s2 and s3

S9 s6 or s7 or s8

S10 s9 and s4 and s5

S11 drunkorexi*

S12 S10 or S11

### LILACS

Search 1

(alcohol$ or drink$) [Words] AND (food$ or overeat$ or obes$ or overweight) [Words] AND (young adult) [Limits]

Search 2

(cluster$ or covariat$) [Words] and (alcohol$ or drink$) [Words] and (young adult) [Limits]
