# Supplementary material for: Non-Pharmacological Interventions to Reduce Unhealthy Eating and Risky Drinking in Young Adults Aged 18–25 Years: A Systematic Review and Meta-Analysis
Source: Nutrients. 2018 Oct 18;10(10):1538. doi: 10.3390/nu10101538 (PMC6213108; doi:10.3390/nu10101538)
Supplement: Supplementary file 1 [file nutrients-10-01538-s001.zip › nutrients-364264-sup/Supplementary Table S2 Intervention content and delivery.docx]

**Supplementary Table S2:** Intervention content and delivery.

| **Reference:** | **Description of Intervention:** | **Theoretical Basis:** | **Intervention Frequency, Length and Duration:** | **Delivery (Mechanism, Medium, Intensity, Fidelity, Providers):** | **Comparator / Control:** |
| --- | --- | --- | --- | --- | --- |
| Ashton et al (2017) | 7 program components: 1) Responsive website that served as a ‘resource library’ housing relevant information and resources, including fact sheets from best practice guidelines, support videos (e.g. short cooking videos and demonstration of Gymstick™ exercises) and recommended mobile applications for improving eating habits, physical activity, reducing alcohol intake or coping with stress; 2) Jawbone™ wearable physical activity tracker with associated mobile phone application (UP app) to assist in goal setting and self-monitoring of key health behaviours; 3) One-hour weekly face to face sessions at the university (11x group based and 1x individual); 4) Personalised food and nutrient report comparing intakes to Australian food and nutrient recommendations. This feedback report was given to participants and discussed in the individualised session (week 3) and used to set personal tailored goals for dietary improvements; 5) Private Facebook discussion group to facilitate social support, send reminders for upcoming face-to-face sessions and send notifications for new material added to the website; 6) Gymstick™ resistance band, for home-based strength training with linked routines available on the website; 7) TEMPlate™ dinner disc to guide main meal portion size for main meal components. | Based on guidance from a community based participatory research model; PRECEDE-PROCEED, as well as theoretical guidelines from an integrated framework of Social Cognitive Theory (SCT) and Self Determination Theory (SDT) | Weekly one-hour group based sessions over 3 months, with 40mins allocated for practical exercise activities focusing on aerobic (e.g. team based recreational games) and strength exercises (e.g. High Intensity Interval Training); ten mins allocated for healthy eating education (e.g. meal planning and meal ideas) and 10mins for helping with stress and well-being, including a mixture of practical (e.g. mindfulness based stress reduction) and theoretical (e.g. problem solving strategies to address key issues i.e. lack of money) components. | Sessions delivered by two male researchers from the same age demographic (one was a qualified P.E. teacher, undertaking a PhD in Education and the other was a PhD candidate in Nutrition and Dietetics). | Waitlist control group: Control participants were asked to continue their usual lifestyle for 3 months and offered the HEYMAN program once follow-up assessments were completed. |
| Epton et al (2014); Cameron et al (2015) | Three theory-based techniques to promote behaviour change: 1) a self-affirmation manipulation to reduce defensive processing of health messages; 2) theory-based messages designed to increase motivation to adopt healthy behaviours; 3) participants prompted to form implementation intentions to help them to translate their intentions into behaviour change. The self-affirmation manipulation was adapted from an existing value-affirmation task and embedded in a ‘profile’ page. Participants were asked to provide details including their name, course, home town and main interests or hobbies, before being presented with a list of eight commonly held personal values (sense of humour, academic achievement, relations with family and friends, social skills, spontaneity, artistic skills or aesthetic appreciation, religion, faith or spirituality, and respect, decency or manners). Participants were asked to select their most important value (or provide their own) and to explain briefly why the value was important to them. The resultant information formed part of the user’s ‘profile’, which was displayed in the banner at the top of all pages of the intervention website that included the participant’s name, the value that they chose and the reason why it was important to them. Participants were then directed to complete short modules on each of the four targeted health behaviours. Theory-based messages were developed to encourage adequate fruit and vegetable intake and regular exercise, and to discourage binge drinking and smoking. For each module, participants were presented with a list of topics that targeted key beliefs from the formative research and instructed to choose one. They were then directed to a webpage containing theory-based messages (i.e., text), videos of students talking about the targeted belief, and links to other related material. After viewing the page, participants had the opportunity to either view another topic or message or proceed to the planner. The planner helped participants to form implementation intentions by asking them to identify (i) a good opportunity to act on their intentions and (ii) a suitable response to their identified opportunity for each of the four targeted health behaviours. Participants were presented with an example of an implementation intention in an if-then format. Spaces were provided for participants to make up to three if-then plans by linking an identified opportunity and appropriate response. Participants were presented with their plan and asked to repeat it to themselves several times. A record of the plan was also automatically emailed to the participant. When all four modules had been completed, participants had access to the full website, containing messages targeting all of the key beliefs from the formative research, links to the planner, saved plans and general health information. All participants completed the modules in the same order as described. | Based on self-affirmation theory, the Theory of Planned Behaviour and implementation intentions); developed on the basis of formative work that identified the key behavioural, normative and control beliefs associated with intentions to perform each of the four health behaviours in new university students. | Unknown length/duration - intervention was accessible from laptops and desktop computers, smart phones, with Twitter feed, Facebook and Google+ pages.  After completing the self-affirmation manipulation, participants had to work through 4 modules and then had open access to the website. Engagement with the digital intervention was recorded, such as no. of visits, number and type of pages visited, the number of implementation intention plans completed. | Web-based | Control group not directed to the four modules or the website. Individuals in the control group were only sent web-based questionnaires. |
| Kypri and McAnally (2005) | All participants completed a web-based questionnaire in the waiting area. Groups A, B, and C entered demographic details and had their blood pressure measured. Participants in groups A and B completed assessments on fruit and veg consumption; alcohol consumption; smoking; physical activity and mental health. Participants in group A only were then presented with feedback. For each behaviour, feedback was presented in terms of: (1) health authority recommendations; (2) social norms and self-comparison, e.g., recommendations to eat at least two servings of fruit and three servings of vegetables per day; that adherence to these guidelines reduces the risk of heart disease, some cancers, and type II diabetes; the percentage of the population of the same age and gender adhering to these recommendations, directly compared to the participant’s level of fruit and vegetable intake. | Not discussed | Single Session | Web-based | Group C: completed a web-based questionnaire, entered demographic details and had their blood pressure measured only. Group C participants were thanked and told they would be contacted by e-mail in 6 weeks to complete a web-based follow-up assessment. |
| Leiva et al (2015) | Two intervention areas:   1. Education for Health: included academic specialists in this area and comprised theoretical alternate workshops on food counselling and healthy lifestyles.   - Introduction to the subject, signing of informed consent and commitment to self-care; completion of questionnaire  - Lifestyles and cardiovascular risk factors  - Benefits of physical activity for health  - Healthy nutrition  - Dietary Guidelines and Nutrition Labelling  Practical workshops: 2 sessions of 90 min each  - Tasting healthy food (with your recipes)  - Learning to cook healthy  Counselling: 2 sessions of 45 min each  - Healthy nutrition  - Physical activity plan according to needs   1. Physical activity: included students graduating with a career in Physical Education in Health. They were offered three weekly sessions of sports-recreational programs spread over 17 weeks.   - Quick walk, entertaining dance, jogging, kayaking, physical conditioning, football, hum, volleyball, outdoor games | Not discussed | 17 weeks.  Education for Health: 5 theoretical sessions lasting 45 minutes each; 2 90-minute practical workshops; 2 45-minute counselling sessions.  Physical activity: 60 minute sessions | Face-to-face; Provider unclear but appears to be degree tutors | No control (pre/post study). |
| Quartiroli and Zizzi (2012) | Participants randomly assigned to one of two intervention conditions by the KBS and received either personalized (PERS) or normative (NORM) feedback. After completion of three surveys, students in the PERS group received personalized feedback, developed by the lead author and delivered through the Knowledge Based System (KBS). The researcher, who analysed all the possible combinations of responses that the system could receive, produced a theory-based paragraph for each combination of responses, which together formed the personalized feedback. The personalized feedback provided the individual with a description of their current behaviours. It also included suggestions to develop, improve, and/or maintain a healthy lifestyle. This personalized feedback was based on Nutritional and Physical Activity Guidelines for Americans found in the scientific literature. It was characterized by its ability to integrate the personal response of the individuals with the contextualized information and the scientific literature. Participants in the NORM group were provided with the same normative feedback not considering their individual responses. This feedback was developed by the researcher and based on the Guidelines for Americans and the scientific literature, and by simply listing the guidelines only. | The theoretical frameworks to make up the questionnaire were the Transtheoretical Model of Change (TTM) and the Theory of Planned Behaviour (TPB). Dillman’s principles of tailored design were used for the development of survey in addition to other health behaviour surveys | Three surveys over an 8 week period. The first administration of the survey was held during the third and fourth weeks of the spring semester of 2011, the second during the seventh, and the third during the tenth week. The survey took approximately 14 minutes to complete. | Web-based | No control. |
| Werch et al (2008) | Fitness Behaviour Image Screen:  Participants in both groups were first asked to complete the Fitness Behavior Screen, a 9-item instrument designed to elicit responses on selected health behaviors and self-images addressed in the consultation and goal plan. The items asked participants about their physical activity, exercise, diet, sleep, stress management habits, gender, and their alcohol and cigarette use, as well as their desire to achieve selected images, using primarily yes and no response items. Responses were used to tailor consultation messages to each participant’s specific health habits.  One-to-one Consultation and Goal Plan: Participants were provided with scripted messages by the fitness specialist using a consultation protocol. The consultation protocol provided tailored content addressing each of the health behaviors in the screen and their relation to salient image achievement. PowerPoint slides were shown at designated points to reinforce key images and health behaviors using colourful text and illustrations during the dialog-based consultation. Illustrations represented a wide range of young adult ages (18–21 years old), ethnic backgrounds, and both genders. At the conclusion of the consult, the fitness specialist provided participants with a 1-page goal plan. The plan included fitness recommendations which reiterated the key points of the consultation and coupled salient images with target behaviours. Students were asked to select at least one goal from each of 4 behavior groups to improve during the next week, including: (1) increase physical activity and exercise, (2) decrease alcohol use, (3) decrease cigarette use, or (4) increase other fitness behaviors (i.e., nutrition, stress management, sleep). | Behaviour Image Model (BIM), supported by prospect theory and related literature on message framing | 25 minutes; Single Session | One-to-one; Face-to-face. Quality of consultation and goal plan implementation was ensured by using a standardized implementation protocol, with randomly selected intervention sessions audio-taped to monitor implementation quality across interventionists. Fitness specialist (trained bachelor's level research staff). All fitness specialists received a 2-day training that included demonstrations, role-playing with other research personnel, feedback from research staff, and take-home practice on how to implement the consultation and goal plan. | Standard care control consisting of commercial health education print materials, distributed as participants presented for appointments with a fitness specialist |
| Werch et al (2007) | Consultation:  Participants first completed a 9-item Fitness Behaviour Screen. The consultation protocol provided tailored content addressing each of the health behaviors in the screen and their relation to salient image achievement. PowerPoint slides were shown at designated points in the consultation to highlight key images and health behaviors using colourful text and illustrations. Consultation plus contract: Participants were administered the same screen, followed by the consultation, and then the contract with calendar log by the fitness specialist.  Contract: Fitness specialist provided participants with a contract and assisted them in completing it. Each contract asked students to select at least one behaviour from each of 4 behaviour groups to improve in the next week, including physical activity/exercise, alcohol misuse, and substance use (cigarette and marijuana use), and an “other health behaviour” category. Participants were then given a 12-week calendar log to check off the behaviour goals they achieved at the end of each day of the week, and were instructed to mail back only the first week of the log to the research office using a self-addressed, stamped envelope. The 3-month (12-week) calendar log was selected to provide additional support and potentially extend the influence of the brief, one-time contract strategy, while not overburdening participants with a long-term commitment. | Behaviour Image Model (BIM), supported by prospect theory and related literature on message framing | Consultation: 25 minutes  Consultation plus contract: Not specified but, added together, consultation plus contract should last approximately 40 minutes.  Contract: 15 minutes  Single Session | Face-to face. Quality of consultation and contract protocol implementation was monitored over time by audio-taping selected sessions. Periodic meetings were held with the fitness specialist to provide feedback regarding protocol compliance, as well as to discuss specific steps to enhance protocol implementation.  Fitness specialist (trained bachelor's level research staff): received a 2-day training that included demonstrations, role-playing with other research personnel, feedback from project staff, and take-home practice on how to implement the screen, consultation, and contract. | No control (three active conditions) |
