# Supplementary material for: Non-Pharmacological Interventions to Reduce Unhealthy Eating and Risky Drinking in Young Adults Aged 18–25 Years: A Systematic Review and Meta-Analysis
Source: Nutrients. 2018 Oct 18;10(10):1538. doi: 10.3390/nu10101538 (PMC6213108; doi:10.3390/nu10101538)
Supplement: Supplementary file 1 [file nutrients-10-01538-s001.zip › nutrients-364264-sup/Supplementary Table S3 Risk of bias assessment for RCTs..docx]

**Supplementary Table S3:** Risk of bias assessment for RCTs**.**

| **Entry** | **Judgement** | **Support for Judgement** |
| --- | --- | --- |
| Ashton et al (2017) | | |
| Random sequence generation (selection bias) | Low risk | Quote: “Participants were randomised by an independent research assistant who had no contact with participants during the trial. The allocation sequence was generated by a computer based random number algorithm (https://www.sealedenvelope.com/simple-randomiser/v1/lists) producing individual group allocation in block lengths of six.” |
| Allocation concealment (selection bias) | Low risk | Quote: “Randomisation codes were stored in a restricted computer folder, which was not accessible by those assessing participants or those participating in data entry for the study. Complete separation was achieved between the research assistant who generated the randomisation sequence, those who concealed allocation and from those involved in implementation of assignments.” |
| Blinding of participants and personnel (performance bias) | Low risk | Quote: “All measurements were performed by trained research assistants who were blinded to group allocation.” |
| Blinding of outcome assessment (detection bias) | Low risk | Quote: “All measurements were performed by trained research assistants who were blinded to group allocation.” |
| Incomplete outcome data addressed (attrition bias) | Low risk | Comment: No missing outcome data; number randomized and followed up clearly stated |
| Selective reporting (reporting bias) | Low risk | Quote: “The trial was registered with the Australian New Zealand Clinical Trials Registry, Number ACTRN12616000350426”. |
| **Overall assessment: Low risk** | | |
| Epton et al (2014); Cameron et al (2015) | | |
| Random sequence generation (selection bias) | Low risk | Quote: “using the random function on SurveyGizmo” (Epton et al)  Quote: “using the random function on LifeGuide” (Cameron et al)  Comment: Kruger et al based on health economics analysis of the Epton et al study cohort. |
| Allocation concealment (selection bias) | Low risk | Quote: “using the random function on SurveyGizmo” (Epton et al)  Quote: “using the random function on LifeGuide” (Cameron et al)  Comment: Probably done  Comment: Kruger et al based on health economics analysis of the Epton et al study cohort. |
| Blinding of participants and personnel (performance bias) | Low risk | Comment: Behavioural intervention rather than pharmaceutical; therefore outcomes unlikely to be affected by blinding; Assumption is yes because randomization generated by computer programme |
| Blinding of outcome assessment (detection bias) | Low risk | Comment: No blinding of outcome assessment, but outcome measurement is not likely to be influenced by lack of blinding (for all outcomes listed) |
| Incomplete outcome data addressed (attrition bias) | Low risk | Quote: “Missing data at 6-months were imputed from the 1-month follow-up data by carrying the last observation forward” (Epton et al; Cameron et al)  Quote: “Missing utility data at either follow-up point was imputed with that individuals’ utility value from the other follow-up point (a combination of last observation carried forward and last observation carried backward)” (Kruger et al)  Comment: Missing data have been imputed using appropriate methods. |
| Selective reporting (reporting bias) | Low risk | Quote: “Full details of the intervention are provided in an earlier paper reporting the study protocol” (Epton et al)  Quote: “The data analysis plan for the repeat trial was the same as for the original trial and as reported in the study protocol” (Cameron et al)  Comment: Kruger et al based on health economics analysis of the Epton et al study cohort.  Comment: Both trials registered with an ISRCTN number  Comment: Per-protocol and intention to treat analysis conducted in both trials |
| **Overall assessment: Low risk** | | |
| Kypri and McAnally (2005) | | |
| Random sequence generation (selection bias) | Low risk | Quote: “Participants were assigned by a computerized random number generator in blocks of 15 (five per trial arm), to ensure approximately equal group sizes in a short recruitment period.” |
| Allocation concealment (selection bias) | Low risk | Quote: “Participants were assigned by a computerized random number generator in blocks of 15 (five per trial arm), to ensure approximately equal group sizes in a short recruitment period.”  Quote: “Allocation concealment was achieved by not informing participants that they were participating in an intervention trial, in accordance with ethical approval. Additionally, the research assistant recruiting participants was not informed of group allocation, which was done by computer.” |
| Blinding of participants and personnel (performance bias) | Low risk | Quote: “Participants were assigned by a computerized random number generator in blocks of 15 (five per trial arm), to ensure approximately equal group sizes in a short recruitment period.”  Quote: “Allocation concealment was achieved by not informing participants that they were participating in an intervention trial, in accordance with ethical approval. Additionally, the research assistant recruiting participants was not informed of group allocation, which was done by computer.” |
| Blinding of outcome assessment (detection bias) | Low risk | Comment: No blinding of outcome assessment, but outcome measurement is not likely to be influenced by lack of blinding (for all outcomes listed) |
| Incomplete outcome data addressed (attrition bias) | Unclear risk | Comment: no reason for missing data provided; number randomized and followed up clearly stated |
| Selective reporting (reporting bias) | Unclear risk | Comment: study protocol unavailable; unclear whether outcomes were pre-specified and reported in the pre-specified way; no trial registration specified. |
| **Overall assessment: Unclear risk** | | |
| Werch et al (2008) | | |
| Random sequence generation (selection bias) | Unclear risk | Quote: “Participants were randomly assigned to one of two treatment arms”  Comment: No further detail. |
| Allocation concealment (selection bias) | Unclear risk | Quote: “Participants were randomly assigned to one of two treatment arms”  Comment: No further detail. |
| Blinding of participants and personnel (performance bias) | Unclear risk | Comment: insufficient information |
| Blinding of outcome assessment (detection bias) | Low risk | Comment: No blinding of outcome assessment, but outcome measurement is not likely to be influenced by lack of blinding (for all outcomes listed) |
| Incomplete outcome data addressed (attrition bias) | High risk | Quote: “Sixteen participants were lost to attrition (5%), with no differences in attrition between treatment groups. Significantly more students who dropped out of the study received mostly B grades (rather than A grades) on their last report card, reported a family alcohol or drug problem, and used marijuana in the past 30 days than those who did not drop out.” |
| Selective reporting (reporting bias) | Unclear risk | Quote: “The university’s institutional review board approved the research protocol prior to implementing the study.”  Comment: Protocol mentioned but not made available; unclear whether outcomes were pre-specified and reported in the pre-specified way; no trial registration specified. |
| **Overall assessment: High risk** | | |
| Werch et al (2007) | | |
| Random sequence generation (selection bias) | Low risk | Quote: “Participants were randomly assigned to treatments as they presented at the clinic by using packets that were pre-randomized in sets to ensure equivalent groups.” |
| Allocation concealment (selection bias) | Unclear risk | Quote: “Participants were randomly assigned to treatments as they presented at the clinic by using packets that were pre-randomized in sets to ensure equivalent groups.”  Comment: assignment packets are described but unclear whether these were sequentially numbered, opaque and sealed. |
| Blinding of participants and personnel (performance bias) | Unclear risk | Comment: insufficient information |
| Blinding of outcome assessment (detection bias) | Low risk | Comment: No blinding of outcome assessment, but outcome measurement is not likely to be influenced by lack of blinding (for all outcomes listed) |
| Incomplete outcome data addressed (attrition bias) | Unclear risk | Quote: “Seven participants were lost to attrition (5%), with attrition distributed across treatment groups”.  Comment: insufficient information on number randomized / numbers followed up / reasons for attrition. |
| Selective reporting (reporting bias) | Unclear risk | Quote: “The university’s institutional review board approved the research protocol prior to implementing the study.  Comment: Protocol mentioned but not made available; unclear whether outcomes were pre-specified and reported in the pre-specified way; no trial registration specified. |
| **Overall assessment: Unclear risk** | | |
