# Supplementary material for: Non-Pharmacological Interventions to Reduce Unhealthy Eating and Risky Drinking in Young Adults Aged 18–25 Years: A Systematic Review and Meta-Analysis
Source: Nutrients. 2018 Oct 18;10(10):1538. doi: 10.3390/nu10101538 (PMC6213108; doi:10.3390/nu10101538)
Supplement: Supplementary file 1 [file nutrients-10-01538-s001.zip › nutrients-364264-sup/Supplementary Table S4 EPHPP quality appraisal for non-randomised or experimental studies.docx]

**Supplementary Table S4:** EPHPP quality appraisal for non-randomised or experimental studies.

| **Entry** | **Judgement** | **Rating** |
| --- | --- | --- |
| Leiva et al (2015) | | |
| Selection bias | Q1 – Very likely  Q2 – 80-100% agreement | Strong |
| Study design | Cohort – described as ‘quasi-experimental’  NO – no randomisation | Moderate |
| Confounders | Q1 – Not applicable (pre/post study)  Q2 – Can’t tell (N/A) | Weak |
| Blinding | Q1 – Can’t tell (N/A)  Q2 – Can’t tell (N/A) | Weak |
| Data collection methods | Q1 – Yes  Q2 – No (not described) | Moderate |
| Withdrawals and drop-outs | Q4 – Yes  Q2 – 80-100% | Strong |
| Intervention integrity | Q1 – 80-100%  Q2 – No  Q3 - No | |
| Analyses | Q1 – Not applicable  Q2 – Not applicable  Q3 – appropriate statistical methods used  Q4 – Not applicable | |
| **Overall assessment: Weak** | | |
| Quartiroli and Zizzi (2012) | | |
| Selection bias | Q1. Very likely  Q2. Less than 60% agreement | Weak |
| Study design | Other – described as pseudo experimental  YES – described as randomised  NO – no details given on method of randomisation | Weak |
| Confounders | Q1 – Can’t tell  Q2 – Can’t tell | Weak |
| Blinding | Q1 – Can’t tell  Q2 - Yes | Weak |
| Data collection methods | Q1 – Can’t tell  Q2 – Can’t tell | Weak |
| Withdrawals and drop-outs | Q1 – No  Q3 – less than 60% | Weak |
| Intervention integrity | Q1 – Less than 60%  Q2 – No  Q3 – Can’t tell | |
| Analyses | Q1 – individual  Q2 – individual  Q3 – appropriate statistical methods used  Q4 – Yes | |
| **Overall assessment: Weak** | | |
